# {2-[(3,5-Dichloro-2-oxidobenzyl­idene)amino-κ^2^ *N*,*O*]-3-methyl­penta­noato-κ*O*}(*N*,*N*′-dimethyl­formamide-κ*O*)copper(II)

**DOI:** 10.1107/S160053680800932X

**Published:** 2008-04-16

**Authors:** Jin Hong Xia, Zheng Liu, Yuan Wang, Xiao Zhen Feng

**Affiliations:** a College of Electronic Engineering, Guilin University of Electronic Technology, Ministry of Education, Guangxi, Guilin 541004, People’s Republic of China; b Key Laboratory of Non-ferrous Metal Materials and Processing Technology, Department of Materials and Chemical Engineering, Guilin University of Technology, Ministry of Education, Guilin 541004, People’s Republic of China

## Abstract

In the title compound, [Cu(C_13_H_13_Cl_2_NO_3_)(C_3_H_7_NO)], the Cu^II^ atom is coordinated in a slightly distorted square-planar geometry by two O atoms and one N atom from the tridentate chiral ligand 2-[(3,5-dichloro-2-oxidobenzyl­idene)amino]-3-methyl­penta­noate and by one O atom from dimethyl­formamide. In the crystal structure, the Cu atom forms contacts with Cl and O atoms of two units (Cu⋯Cl and Cu⋯O = 3.401 and 2.947 Å, respectively), thereby forming an approximately octa­hedral arrangement. A three-dimensional network is constructed through Cl⋯Cu, O⋯Cu, Cl⋯Cl contacts and C—H⋯O hydrogen bonds.

## Related literature

For Schiff base complexes containing amino acids, see: Garcia-Raso *et al.* (1996 ▶); Dawes *et al.* (1982 ▶); Laurent *et al.* (1982 ▶); Zhang *et al*. (2006 ▶). For related literature, see: Cohen *et al.* (1964 ▶); Garcia-Orozco *et al.* (2002 ▶); Hu & Englert (2006 ▶); Royles & Sherrington (2000 ▶); Subramanian *et al.* (2000 ▶); Zaman *et al.* (2004 ▶); Zordan *et al.* (2005 ▶).
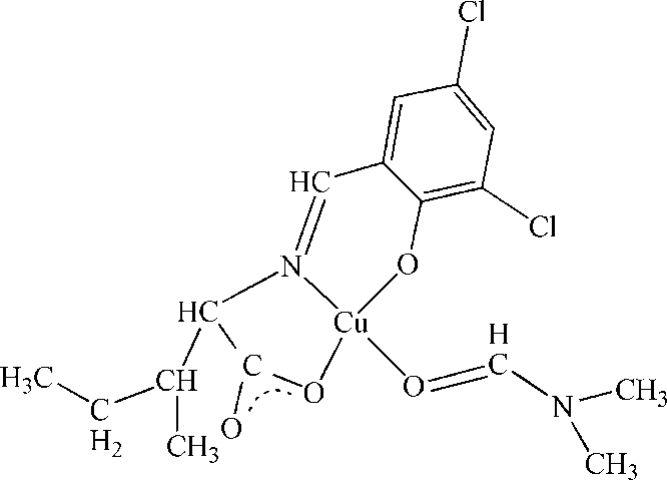


## Experimental

### 

#### Crystal data

[Cu(C_13_H_13_Cl_2_NO_3_)(C_3_H_7_NO)]
*M* *_r_* = 438.78
Orthorhombic, 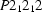

*a* = 11.671 (2) Å
*b* = 27.465 (3) Å
*c* = 5.8890 (18) Å
*V* = 1887.7 (7) Å^3^
*Z* = 4
Mo *K*α radiation
μ = 1.46 mm^−1^
*T* = 298 (2) K
0.43 × 0.15 × 0.13 mm

#### Data collection

Bruker SMART CCD area-detector diffractometer
Absorption correction: multi-scan (*SADABS*; Sheldrick, 1996 ▶) *T*_min_ = 0.572, *T*_max_ = 0.833
9274 measured reflections
3260 independent reflections
2288 reflections with *I* > 2σ(*I*)
*R*_int_ = 0.086

#### Refinement

*R*[*F* ^2^ > 2σ(*F* ^2^)] = 0.095
*wR*(*F* ^2^) = 0.242
*S* = 1.05
3260 reflections
226 parameters
H-atom parameters constrained
Δρ_max_ = 0.54 e Å^−3^
Δρ_min_ = −1.08 e Å^−3^
Absolute structure: Flack (1983 ▶), with 1915 Friedel pairs
Flack parameter: 0.11 (6)

### 

Data collection: *SMART* (Bruker, 2001 ▶); cell refinement: *SAINT* (Bruker, 2001 ▶); data reduction: *SAINT*; program(s) used to solve structure: *SHELXS97* (Sheldrick, 2008 ▶); program(s) used to refine structure: *SHELXL97* (Sheldrick, 2008 ▶); molecular graphics: *SHELXTL* (Sheldrick, 2008 ▶); software used to prepare material for publication: *SHELXTL*.

## Supplementary Material

Crystal structure: contains datablocks global, I. DOI: 10.1107/S160053680800932X/gw2039sup1.cif

Structure factors: contains datablocks I. DOI: 10.1107/S160053680800932X/gw2039Isup2.hkl

Additional supplementary materials:  crystallographic information; 3D view; checkCIF report

## Figures and Tables

**Table:** 

Cu1—O3	1.872 (9)
Cu1—O1	1.905 (9)
Cu1—O4	1.920 (9)
Cu1—N1	1.925 (10)

**Table:** 

O3—Cu1—O1	169.2 (4)
O3—Cu1—O4	92.5 (4)
O1—Cu1—O4	90.5 (3)
O3—Cu1—N1	94.2 (4)
O1—Cu1—N1	82.7 (4)
O4—Cu1—N1	173.1 (4)

**Table 2:** Hydrogen-bond geometry (Å, °)

*D*—H⋯*A*	*D*—H	H⋯*A*	*D*⋯*A*	*D*—H⋯*A*
C15—H15*B*⋯O2^i^	0.96	2.31	3.19 (2)	150

Symmetry code: (i) 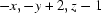.

**Fig. 1.**
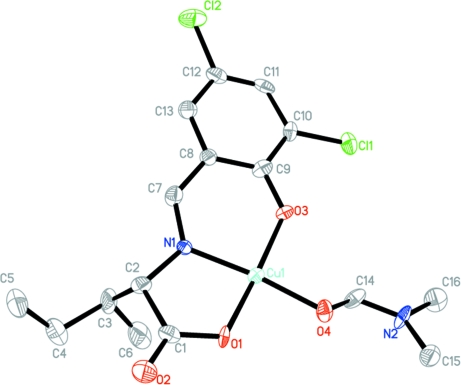
The asymmetric unit of (I), showing 30% probability displacement ellipsoids. Carbon-bound H atoms have been omitted.

**Fig. 2.**
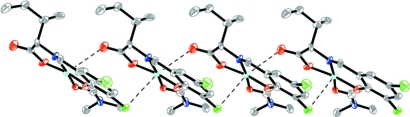
The interactions of Cl···Cu and O···Cu in the asymmetric unit one-dimensional chains.

**Fig. 3.**
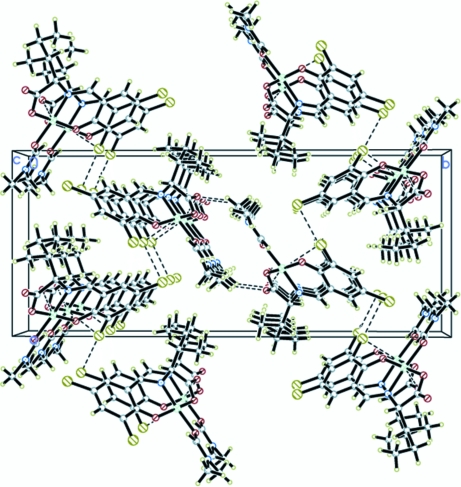
Three-dimensional network of (I), broken line showing Short Cl–Cl, Cl···Cu and O···Cu contacts, C–H···O hydrogen bonds.

## supplementary crystallographic information

### Comment

Schiff bases is a kind of very important compounds in coordination chemistry. And Schiff base complexes have been increasing interest because of their antivial,anticancer and antibacterial activities.Just as the title compound, it's amino acid salicylicaldehyde of halogen substituent Schiff base. Meanwhile,we find some unusual bonds which look like hydrogen bond in this halogenated compound.

In (I),the Cu^II^ atom is coordinated by two O atoms, one N atom, which come from one tridentate ligand 2-[(3,5-dichloro-2-oxidobenzylidene)amino]-3-methylpentanoate and one O atom from *N*,*N*-Dimethyl-formamide, forming a slightly distorted planar square geometry (Fig. 1). In the unit one-dimensional chains, the distorted planar square with Cl and O which above or below of it form an approximately "octahedral". The weak interaction length of Cl–Cu and O–Cu is 3.401 Å. and 2.947 Å. These can be seen the reasults of Jahn-Teller effect. (Garcia-Orozco *et al.*, 2002) People have interest in packing arrangements of halogenated compounds date back to what Schmidt called the 'chloro effect', where the presence of chloro substituents on aromatic compounds frequently arise from stacking arrangements with a short (*ca* 4 Å) crystallographic axis (Cohen *et al.*, 1964; Zaman *et al.*, 2004; Zordan *et al.*, 2005). The title compound contains the dichloride ligand 2-[(3,5-dichloro-2-oxidobenzylidene)amino]-3-methylpentanoate, with two Cl atoms accessible at the periphery of the ligand. The three-dimensional network of (I) through short Cl–Cu, Cl–Cl, O–Cu contacts and C–H···O hydrogen bonds.(Fig.3). The weak interaction length of Cl–Cl is 3.349 Å. The final position parameters of the nonhydrogen atoms are given in Table 1. The selected bond lengths and bond angles are listed in Table 2.

### Experimental

The title compound was produced from aqueous solution of copper chloride and A ethanol solution of (*E*)-2-(3,5-dichloro-2-hydroxybenzylideneamino)-3 -methylpentanoic acid with vapour volatilization of *N*,*N*-Dimethyl-formamide at room temperature.

### Crystal data

**Table:**

[Cu(C_13_H_13_Cl_2_NO_3_)(C_3_H_7_NO)]	*D*_x_ = 1.544 Mg m^−^^3^
*M**_r_* = 438.78	Mo *K*α radiation λ = 0.71073 Å
Orthorhombic, *P*2_1_2_1_2	Cell parameters from 1991 reflections
*a* = 11.671 (2) Å	θ = 2.3–20.3º
*b* = 27.465 (3) Å	µ = 1.46 mm^−^^1^
*c* = 5.8890 (18) Å	*T* = 298 (2) K
*V* = 1887.7 (7) Å^3^	Block, blue
*Z* = 4	0.43 × 0.15 × 0.13 mm
*F*_000_ = 900	

### Data collection

**Table:**

Bruker SMART CCD area-detector diffractometer	3260 independent reflections
Radiation source: fine-focus sealed tube	2288 reflections with *I* > 2σ(*I*)
Monochromator: graphite	*R*_int_ = 0.087
*T* = 298(2) K	θ_max_ = 25.0º
φ and ω scans	θ_min_ = 1.5º
Absorption correction: multi-scan(SADABS; Sheldrick, 1996)	*h* = −9→13
*T*_min_ = 0.572, *T*_max_ = 0.833	*k* = −32→32
9274 measured reflections	*l* = −6→6

### Refinement

**Table:**

Refinement on *F*^2^	Hydrogen site location: inferred from neighbouring sites
Least-squares matrix: full	H-atom parameters constrained
*R*[*F*^2^ > 2σ(*F*^2^)] = 0.095	*w* = 1/[σ^2^(*F*_o_^2^) + (0.1179*P*)^2^ + 7.0901*P*] where *P* = (*F*_o_^2^ + 2*F*_c_^2^)/3
*wR*(*F*^2^) = 0.242	(Δ/σ)_max_ = 0.001
*S* = 1.05	Δρ_max_ = 0.54 e Å^−^^3^
3260 reflections	Δρ_min_ = −1.08 e Å^−^^3^
226 parameters	Extinction correction: none
Primary atom site location: structure-invariant direct methods	Absolute structure: Flack (1983), 1310 Friedel pairs
Secondary atom site location: difference Fourier map	Flack parameter: 0.11 (6)

### Special details

**Table:**

Geometry. All e.s.d.'s (except the e.s.d. in the dihedral angle between two l.s. planes) are estimated using the full covariance matrix. The cell e.s.d.'s are taken into account individually in the estimation of e.s.d.'s in distances, angles and torsion angles; correlations between e.s.d.'s in cell parameters are only used when they are defined by crystal symmetry. An approximate (isotropic) treatment of cell e.s.d.'s is used for estimating e.s.d.'s involving l.s. planes.
Refinement. Refinement of *F*^2^ against ALL reflections. The weighted *R*-factor *wR* and goodness of fit *S* are based on *F*^2^, conventional *R*-factors *R* are based on *F*, with *F* set to zero for negative *F*^2^. The threshold expression of *F*^2^ > σ(*F*^2^) is used only for calculating *R*-factors(gt) *etc*. and is not relevant to the choice of reflections for refinement. *R*-factors based on *F*^2^ are statistically about twice as large as those based on *F*, and *R*- factors based on ALL data will be even larger.

### Fractional atomic coordinates and isotropic or equivalent isotropic displacement parameters (Å^2^)

**Table:**

	*x*	*y*	*z*	*U*_iso_*/*U*_eq_	
Cu1	0.13255 (12)	0.88233 (5)	0.7191 (3)	0.0369 (4)	
Cl1	0.0068 (3)	0.79870 (13)	0.0644 (6)	0.0481 (9)	
Cl2	0.2946 (3)	0.65124 (12)	0.2389 (8)	0.0671 (12)	
N1	0.2606 (8)	0.8445 (3)	0.8257 (17)	0.033 (2)	
N2	−0.1115 (10)	0.9551 (4)	0.3728 (17)	0.043 (3)	
O1	0.1585 (7)	0.9174 (3)	0.9935 (17)	0.046 (2)	
O2	0.2565 (10)	0.9193 (4)	1.3185 (19)	0.063 (3)	
O3	0.0957 (7)	0.8388 (3)	0.4856 (18)	0.044 (2)	
O4	0.0063 (7)	0.9243 (3)	0.6463 (15)	0.039 (2)	
C1	0.2446 (12)	0.9026 (5)	1.125 (2)	0.038 (3)	
C2	0.3198 (12)	0.8638 (4)	1.029 (2)	0.035 (3)	
H2	0.3294	0.8377	1.1411	0.042*	
C3	0.4373 (11)	0.8833 (7)	0.960 (3)	0.059 (4)	
H3	0.4762	0.8584	0.8687	0.071*	
C4	0.5115 (15)	0.8947 (7)	1.168 (3)	0.067 (5)	
H4A	0.5874	0.9039	1.1162	0.080*	
H4B	0.4789	0.9226	1.2457	0.080*	
C5	0.5232 (16)	0.8529 (7)	1.338 (3)	0.077 (5)	
H5A	0.4651	0.8559	1.4523	0.115*	
H5B	0.5974	0.8542	1.4076	0.115*	
H5C	0.5145	0.8224	1.2600	0.115*	
C6	0.4285 (13)	0.9300 (7)	0.818 (3)	0.077 (6)	
H6A	0.3701	0.9261	0.7044	0.116*	
H6B	0.5007	0.9363	0.7453	0.116*	
H6C	0.4092	0.9568	0.9150	0.116*	
C7	0.2818 (10)	0.7995 (4)	0.755 (2)	0.040 (3)	
H7	0.3322	0.7813	0.8426	0.047*	
C8	0.2357 (11)	0.7757 (4)	0.557 (2)	0.034 (3)	
C9	0.1437 (12)	0.7977 (4)	0.428 (2)	0.041 (3)	
C10	0.1061 (8)	0.7732 (4)	0.235 (2)	0.031 (3)	
C11	0.1625 (11)	0.7281 (4)	0.181 (2)	0.043 (3)	
H11	0.1418	0.7123	0.0474	0.051*	
C12	0.2427 (10)	0.7075 (5)	0.310 (3)	0.043 (3)	
C13	0.2807 (11)	0.7309 (5)	0.495 (3)	0.044 (3)	
H13	0.3380	0.7170	0.5836	0.053*	
C14	−0.0233 (13)	0.9290 (4)	0.448 (3)	0.048 (4)	
H14	0.0203	0.9128	0.3393	0.058*	
C15	−0.1907 (13)	0.9786 (5)	0.540 (3)	0.053 (4)	
H15A	−0.2402	0.9544	0.6047	0.080*	
H15B	−0.2361	1.0028	0.4636	0.080*	
H15C	−0.1467	0.9938	0.6579	0.080*	
C16	−0.1495 (16)	0.9579 (7)	0.135 (3)	0.063 (5)	
H16A	−0.0882	0.9478	0.0366	0.094*	
H16B	−0.1709	0.9908	0.0995	0.094*	
H16C	−0.2143	0.9368	0.1129	0.094*	

### Atomic displacement parameters (Å^2^)

**Table:**

	*U*^11^	*U*^22^	*U*^33^	*U*^12^	*U*^13^	*U*^23^
Cu1	0.0356 (7)	0.0313 (6)	0.0437 (9)	0.0098 (6)	−0.0076 (7)	−0.0074 (7)
Cl1	0.0389 (18)	0.061 (2)	0.044 (2)	−0.0044 (16)	−0.0118 (16)	0.0061 (17)
Cl2	0.081 (3)	0.0460 (18)	0.074 (3)	−0.0011 (17)	0.018 (3)	−0.019 (2)
N1	0.039 (5)	0.030 (5)	0.028 (6)	0.009 (4)	−0.013 (5)	−0.012 (4)
N2	0.044 (7)	0.055 (7)	0.030 (6)	0.032 (6)	0.002 (5)	0.011 (5)
O1	0.024 (5)	0.049 (5)	0.066 (7)	0.017 (4)	−0.021 (4)	−0.005 (5)
O2	0.069 (7)	0.066 (7)	0.055 (7)	0.012 (6)	−0.009 (6)	−0.021 (6)
O3	0.040 (5)	0.024 (4)	0.069 (7)	0.009 (4)	−0.012 (4)	−0.010 (4)
O4	0.026 (4)	0.060 (6)	0.031 (5)	0.004 (4)	0.004 (4)	−0.002 (4)
C1	0.032 (7)	0.042 (7)	0.041 (8)	−0.008 (6)	0.012 (6)	−0.014 (6)
C2	0.051 (8)	0.034 (6)	0.020 (6)	0.007 (5)	0.006 (6)	−0.004 (5)
C3	0.022 (7)	0.086 (11)	0.068 (10)	0.020 (8)	−0.006 (7)	−0.017 (11)
C4	0.054 (9)	0.091 (13)	0.056 (10)	−0.008 (9)	−0.011 (8)	−0.011 (9)
C5	0.063 (11)	0.091 (13)	0.076 (13)	0.003 (10)	−0.028 (10)	−0.010 (10)
C6	0.031 (8)	0.111 (14)	0.091 (14)	−0.005 (8)	0.008 (9)	−0.028 (13)
C7	0.040 (7)	0.049 (7)	0.031 (8)	0.014 (5)	0.001 (6)	−0.007 (7)
C8	0.040 (7)	0.032 (6)	0.030 (7)	0.002 (5)	−0.004 (6)	−0.002 (5)
C9	0.048 (8)	0.034 (6)	0.041 (8)	0.008 (6)	0.006 (7)	0.001 (6)
C10	0.022 (6)	0.043 (6)	0.029 (7)	0.001 (4)	0.004 (5)	0.012 (6)
C11	0.051 (8)	0.031 (6)	0.046 (9)	−0.021 (6)	−0.002 (7)	−0.004 (6)
C12	0.030 (6)	0.042 (7)	0.058 (9)	−0.006 (6)	0.016 (7)	−0.025 (7)
C13	0.026 (7)	0.039 (7)	0.067 (10)	0.000 (5)	0.005 (6)	−0.006 (7)
C14	0.059 (9)	0.029 (7)	0.056 (10)	0.015 (6)	0.005 (8)	0.013 (7)
C15	0.052 (9)	0.055 (8)	0.053 (10)	0.012 (7)	−0.008 (8)	−0.006 (7)
C16	0.059 (11)	0.081 (11)	0.048 (10)	0.004 (9)	0.015 (8)	−0.010 (8)

### Geometric parameters (Å, °)

**Table:**

Cu1—O3	1.872 (9)	C5—H5A	0.9600
Cu1—O1	1.905 (9)	C5—H5B	0.9600
Cu1—O4	1.920 (9)	C5—H5C	0.9600
Cu1—N1	1.925 (10)	C6—H6A	0.9600
Cl1—C10	1.685 (11)	C6—H6B	0.9600
Cl2—C12	1.712 (12)	C6—H6C	0.9600
N1—C7	1.328 (14)	C7—C8	1.442 (17)
N1—C2	1.482 (16)	C7—H7	0.9300
N2—C14	1.329 (17)	C8—C13	1.384 (16)
N2—C16	1.473 (19)	C8—C9	1.444 (17)
N2—C15	1.497 (18)	C9—C10	1.395 (17)
O1—C1	1.333 (17)	C10—C11	1.438 (17)
O2—C1	1.233 (16)	C11—C12	1.333 (19)
O3—C9	1.306 (14)	C11—H11	0.9300
O4—C14	1.225 (17)	C12—C13	1.341 (19)
C1—C2	1.493 (17)	C13—H13	0.9300
C2—C3	1.53 (2)	C14—H14	0.9300
C2—H2	0.9800	C15—H15A	0.9600
C3—C4	1.53 (2)	C15—H15B	0.9600
C3—C6	1.53 (3)	C15—H15C	0.9600
C3—H3	0.9800	C16—H16A	0.9600
C4—C5	1.53 (2)	C16—H16B	0.9600
C4—H4A	0.9700	C16—H16C	0.9600
C4—H4B	0.9700		
			
O3—Cu1—O1	169.2 (4)	C3—C6—H6A	109.5
O3—Cu1—O4	92.5 (4)	C3—C6—H6B	109.5
O1—Cu1—O4	90.5 (3)	H6A—C6—H6B	109.5
O3—Cu1—N1	94.2 (4)	C3—C6—H6C	109.5
O1—Cu1—N1	82.7 (4)	H6A—C6—H6C	109.5
O4—Cu1—N1	173.1 (4)	H6B—C6—H6C	109.5
C7—N1—C2	120.0 (10)	N1—C7—C8	127.5 (11)
C7—N1—Cu1	122.9 (8)	N1—C7—H7	116.3
C2—N1—Cu1	115.7 (7)	C8—C7—H7	116.3
C14—N2—C16	125.3 (13)	C13—C8—C7	118.2 (11)
C14—N2—C15	119.5 (12)	C13—C8—C9	121.1 (12)
C16—N2—C15	114.6 (11)	C7—C8—C9	120.7 (10)
C1—O1—Cu1	117.4 (7)	O3—C9—C10	119.5 (11)
C9—O3—Cu1	130.0 (9)	O3—C9—C8	123.1 (11)
C14—O4—Cu1	119.5 (8)	C10—C9—C8	117.4 (10)
O2—C1—O1	120.6 (13)	C9—C10—C11	116.8 (10)
O2—C1—C2	123.3 (14)	C9—C10—Cl1	120.2 (9)
O1—C1—C2	116.1 (11)	C11—C10—Cl1	122.8 (10)
N1—C2—C1	106.7 (11)	C12—C11—C10	124.2 (12)
N1—C2—C3	109.0 (10)	C12—C11—H11	117.9
C1—C2—C3	112.3 (11)	C10—C11—H11	117.9
N1—C2—H2	109.6	C11—C12—C13	119.5 (12)
C1—C2—H2	109.6	C11—C12—Cl2	119.4 (11)
C3—C2—H2	109.6	C13—C12—Cl2	121.1 (12)
C2—C3—C4	111.3 (13)	C12—C13—C8	120.8 (13)
C2—C3—C6	112.3 (11)	C12—C13—H13	119.6
C4—C3—C6	107.6 (15)	C8—C13—H13	119.6
C2—C3—H3	108.5	O4—C14—N2	126.3 (14)
C4—C3—H3	108.5	O4—C14—H14	116.9
C6—C3—H3	108.5	N2—C14—H14	116.9
C5—C4—C3	114.9 (15)	N2—C15—H15A	109.5
C5—C4—H4A	108.5	N2—C15—H15B	109.5
C3—C4—H4A	108.5	H15A—C15—H15B	109.5
C5—C4—H4B	108.5	N2—C15—H15C	109.5
C3—C4—H4B	108.5	H15A—C15—H15C	109.5
H4A—C4—H4B	107.5	H15B—C15—H15C	109.5
C4—C5—H5A	109.5	N2—C16—H16A	109.5
C4—C5—H5B	109.5	N2—C16—H16B	109.5
H5A—C5—H5B	109.5	H16A—C16—H16B	109.5
C4—C5—H5C	109.5	N2—C16—H16C	109.5
H5A—C5—H5C	109.5	H16A—C16—H16C	109.5
H5B—C5—H5C	109.5	H16B—C16—H16C	109.5
			
O3—Cu1—N1—C7	−10.6 (11)	C6—C3—C4—C5	176.2 (14)
O1—Cu1—N1—C7	159.0 (11)	C2—N1—C7—C8	−177.5 (12)
O3—Cu1—N1—C2	−177.3 (8)	Cu1—N1—C7—C8	16.4 (19)
O1—Cu1—N1—C2	−7.7 (8)	N1—C7—C8—C13	171.3 (12)
O3—Cu1—O1—C1	74 (2)	N1—C7—C8—C9	−10 (2)
O4—Cu1—O1—C1	180.0 (9)	Cu1—O3—C9—C10	−175.0 (8)
N1—Cu1—O1—C1	0.4 (9)	Cu1—O3—C9—C8	4.8 (19)
O1—Cu1—O3—C9	−72 (2)	C13—C8—C9—O3	177.1 (12)
O4—Cu1—O3—C9	−177.7 (11)	C7—C8—C9—O3	−2(2)
N1—Cu1—O3—C9	0.7 (12)	C13—C8—C9—C10	−3.1 (18)
O3—Cu1—O4—C14	−34.2 (11)	C7—C8—C9—C10	178.0 (11)
O1—Cu1—O4—C14	156.2 (11)	O3—C9—C10—C11	180.0 (11)
Cu1—O1—C1—O2	−170.2 (10)	C8—C9—C10—C11	0.1 (16)
Cu1—O1—C1—C2	7.0 (14)	O3—C9—C10—Cl1	4.9 (16)
C7—N1—C2—C1	−154.7 (12)	C8—C9—C10—Cl1	−175.0 (9)
Cu1—N1—C2—C1	12.4 (12)	C9—C10—C11—C12	3.9 (18)
C7—N1—C2—C3	83.7 (14)	Cl1—C10—C11—C12	178.8 (10)
Cu1—N1—C2—C3	−109.1 (11)	C10—C11—C12—C13	−5(2)
O2—C1—C2—N1	164.9 (13)	C10—C11—C12—Cl2	174.6 (9)
O1—C1—C2—N1	−12.2 (15)	C11—C12—C13—C8	2(2)
O2—C1—C2—C3	−75.7 (17)	Cl2—C12—C13—C8	−177.8 (10)
O1—C1—C2—C3	107.2 (13)	C7—C8—C13—C12	−178.7 (12)
N1—C2—C3—C4	−169.2 (13)	C9—C8—C13—C12	2(2)
C1—C2—C3—C4	72.8 (17)	Cu1—O4—C14—N2	177.0 (11)
N1—C2—C3—C6	70.2 (15)	C16—N2—C14—O4	−176.1 (15)
C1—C2—C3—C6	−47.9 (17)	C15—N2—C14—O4	−5(2)
C2—C3—C4—C5	52.8 (19)		

### Hydrogen-bond geometry (Å, °)

**Table:**

*D*—H···*A*	*D*—H	H···*A*	*D*···*A*	*D*—H···*A*
C15—H15B···O2^i^	0.96	2.31	3.19 (2)	150

Symmetry codes: (i) −*x*, −*y*+2, *z*−1.
